# A mitochondrial mutator plasmid that causes senescence under dietary restricted conditions

**DOI:** 10.1186/1471-2156-8-9

**Published:** 2007-04-02

**Authors:** Marc FPM Maas, Rolf F Hoekstra, Alfons JM Debets

**Affiliations:** 1 Centre de Génétique Moléculaire, Centre Nationale de la Recherche Scientifique, 1 Avenue de la Terrasse, Gif-sur-Yvette, France; 2 Laboratorium voor Erfelijkheidsleer, Wageningen Universiteit, Arboretumlaan 4, Wageningen, The Netherlands

## Abstract

**Background:**

Calorie or dietary restriction extends life span in a wide range of organisms including the filamentous fungus *Podospora anserina*. Under dietary restricted conditions, *P. anserina *isolates are several-fold longer lived. This is however not the case in isolates that carry one of the pAL2-1 homologous mitochondrial plasmids.

**Results:**

We show that the pAL2-1 homologues act as 'insertional mutators' of the mitochondrial genome, which may explain their negative effect on life span extension. Sequencing revealed at least fourteen unique plasmid integration sites, of which twelve were located within the mitochondrial genome and two within copies of the plasmid itself. The plasmids were able to integrate in their entirety, via a non-homologous mode of recombination. Some of the integrated plasmid copies were truncated, which probably resulted from secondary, post-integrative, recombination processes. Integration sites were predominantly located within and surrounding the region containing the mitochondrial rDNA loci.

**Conclusion:**

We propose a model for the mechanism of integration, based on innate modes of mtDNA recombination, and discuss its possible link with the plasmid's negative effect on dietary restriction mediated life span extension.

## Background

Calorie or dietary restriction (DR) extends life span in a wide range of organisms. This has been known for well over half a century, but the exact mechanisms behind this complex polygenic trait have yet to be revealed. In recent years several important nutrient-sensitive cellular signalling pathways have been discovered, which are at least partially conserved between organisms, *e.g*. the insulin/IGF-1-like and TOR (target of rapamycin) mediated signalling pathways [1-3]. These are involved in adjusting cellular metabolism to nutrient availability. Mutations in them tend to increase longevity and enhance cellular responses to various types of stress, *e.g*. oxidative stress and heat stress. At the proximate level DR thus appears to act by adjusting cellular metabolism to a mode set for survival.

From an evolutionary point of view, the life span extending response to DR can be seen as an adaptation that allows aging organisms to postpone reproduction, and survive unfavourable periods of time [4]. This response may be expected in all aging organisms, including the select subset of filamentous fungi to which *Podospora anserina *belongs. Under regular conditions, *P. anserina *isolates have a replicative life span of two to three weeks [5,6]. They reach a colony diameter of approximately twenty to thirty centimetres, after which their growth irreversibly stops. At 100-fold reduced glucose levels however, they postpone reproduction and live up to five-fold longer [7]. This is associated with higher growth rates, presumably reflecting a type of 'searching' behaviour, resulting in disproportionately larger colony sizes. The degree of life span extension that can be induced by DR in *P. anserina *far exceeds that induced by DR in most other systems, perhaps an evolutionary adaptation that characterizes sessile organisms. Among natural *P. anserina *isolates, its main source of variation is the presence or absence of pAL2-1 homologous mitochondrial plasmids: Whereas plasmid-free isolates respond adequately to DR, plasmid carrying isolates barely do so [7]. The plasmid is sometimes lost in the sexual cycle though, and plasmid-carrying isolates can thus be 'cured'. These 'cured' isolates respond adequately to DR [7], suggesting a causal relationship between the presence of the plasmids and the abrogation of life span extension under DR conditions.

PAL2-1 is a mitochondrial plasmid of the 'invertron'-type [8]. It is the only one of its kind that has thus far been found in *P. anserina*. Invertron-type plasmids are characterized by large terminal inverted repeats (TIRs), as also found in transposable elements, bacteriophages and adenoviruses [9]. Most of them appear to have no substantial phenotypic effect on their host, and invertron-type plasmids are hence often considered to be relatively innocuous molecular parasites. Typically they encode but the bare essential needed for their own replication, including two single subunit (DNA and RNA) polymerases and a terminal protein. The latter is covalently bound to the 5' terminus of the plasmid and serves as a primer for replication. It may operate via a 'snap back' mechanism similar to that involved in adenovirus replication [10]. Replication of the plasmids presumably occurs by a strand-displacement mechanism, again similar to that involved in adenovirus replication [9].

Invertron-type plasmids have also been found in other fungi, *e.g*. in the related ascomycetous genus Neurospora, of which species normally do not senesce. There, like pAL2-1 under DR conditions, the plasmids are associated with a senescence or senescence-like phenomenon [11-17]. The Neurospora senescence plasmids, called pKALILO and pMARANHAR, are able to integrate into the mitochondrial genomes of their hosts. Neither the mode nor the specificity by which they do so is however formally known. The integration mechanism of the more intensively studied plasmid of these two, pKALILO, allegedly involves short sequence homology (SSH) [14]. Based on sequence data alone, the integration of pKALILO initially appeared to be specific for a region within the mitochondrial *rnl *gene [12,13]. Based on more extensive RFLP analysis however, integration later appeared to be random [15]. For unknown reasons, mtDNA molecules that carry an integrated plasmid copy gradually replace the wild-type molecules until the latter are hardly or no longer detectable [15]. The isolates that carry them concomitantly show a wide range of physiological aberrations [11]. Cytological features of senescent cells include extensive vacuolization, swelling and even lysis, perhaps indicating a form of systemic necrosis [16]. Typical features of apoptosis, like cell shrinkage, chromatin condensation and nucleosomal DNA fragmentation have not been observed. Along with the fact that it spreads infectiously [6,17], this clearly indicates that 'plasmid-based' senescence is not part of the normal developmental program, and probably best viewed as a lethal disease.

We surmised that the action of the pAL2-1 homologues would be similar to that of the invertron-type plasmids from Neurospora: *i.e*. integrating into the mitochondrial genome, thereby interfering with mitochondrial function and the response to DR. We tested for the presence of integrated copies of pAL2-1 homologues under regular and under DR conditions. Integrated copies were absent in young cultures, but consistently present in senescent cultures of all the pAL2-1 homologue carrying isolates from our collection. Sequencing revealed 14 unique integration sites of the plasmid, of which twelve were located within the mitochondrial genome and two within copies of the plasmid itself. The plasmids were able to integrate in their entirety, and this did not necessarily involve sequence homology with the mtDNA target site. Some of the integrated plasmid copies were truncated, but this likely resulted from post-integrative recombination processes. Furthermore, integration sites were distributed in a non-random fashion. They were predominantly located within and surrounding the region containing the mitochondrial rDNA loci, supporting the initial inferences from the pKALILO papers [12,13]. We propose a model for the mechanism of integration, based on innate modes of mtDNA recombination, and discuss its possible link with the plasmid's negative effect on DR mediated life span extension.

## Results

### PAL2-1 homologues abrogate the life span extending response to dietary restriction

**Figure 1:**
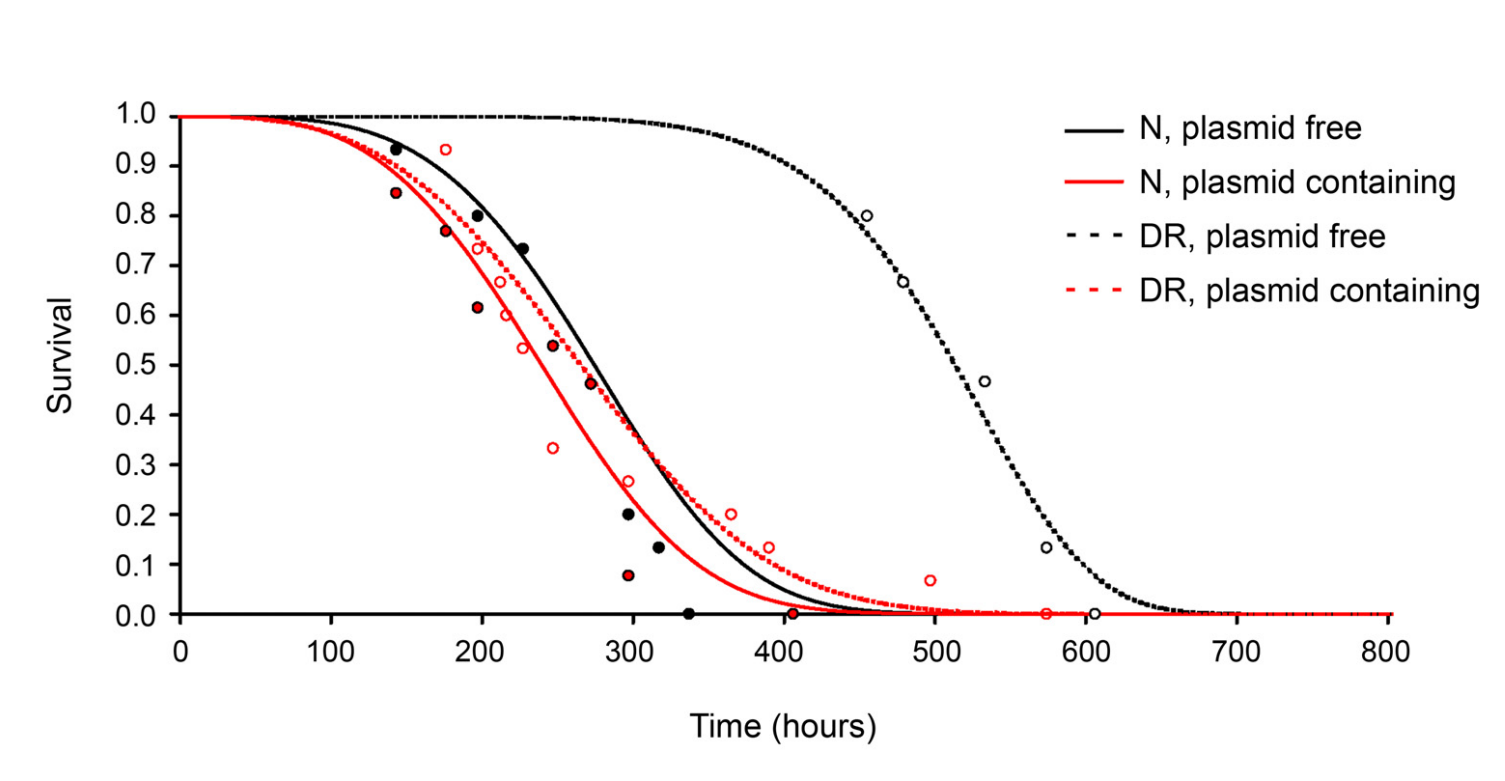
**PAL2-1 homologous mitochondrial plasmids abrogate the life span extending response to dietary restriction**. Survival of near-isogenic lines derived from strain *Wa32*, with (red) and without (black) the pAL2-1 homologous plasmid pWa32-1, under regular (N, filled circles) and glucose-restricted (DR, open circles) conditions. As also previously shown [7], plasmid-carrying isolates respond less adequate to dietary restriction.

We have previously shown that *Podospora anserina *responds to glucose restriction in a highly strain-dependent manner [7]. On semi-synthetic media with 100-fold reduced glucose content, Podospora isolates live up to five-fold longer. This is however not the case in pAL2-1 homologue carrying isolates. Though plasmid containing isolates, like plasmid-free isolates, respond to DR by increased growth rates, they do not or barely respond to DR in a life span extending manner. Near-isogenic lines were constructed with and without plasmids, and this allowed us to separate the effect of the plasmids themselves and any possible effect of the host genetic background [7]. As also illustrated in Figure 1, the pAL2-1 homologous plasmids thus have a pronounced negative effect on life span under dietary restricted conditions.

### Analysis of plasmid integration sites using ST-PCR-based 'plasmid-tagging'

The prototypic plasmid pAL2-1 was first discovered in a long-lived derivative of laboratory strain *A *[18], which purportedly carried two copies of it integrated into the mitochondrial genome. One of these plasmid copies had integrated into the third intron of the mitochondrial *cytb *gene [18-20]. The other one was never characterized. We recently isolated a long-lived derivative of natural isolate *Wa32*. Also this strain (*Wa32-LL*) carried a plasmid copy integrated into the mitochondrial genome [21]. The latter copy was located in the region upstream of the *nd2 *gene. Here we show that the integration of pAL2-1 and/or its homologues is not limited to these two isolated cases in long-lived strains. It occurs readily in senescent cultures as well, and may hence be the reason why DR mediated life span extension is abrogated in plasmid carrying isolates.

As an initial screen, we performed Southern analyses of total DNA from young and old cultures of the previously described pAL2-1 homologue carrying isolates [6,7]. In young cultures the plasmids were present as autonomously replicating elements only, as also previously described [6]. In older cultures however they appeared to integrate. This was the case both under regular and under DR conditions (Southern data not shown). Southern analyses are relatively time consuming, and the data obtained from them is often difficult to interpret due to the many possibilities of post-integrative recombination events. We therefore devised a rapid and unbiased method by which plasmid/mtDNA recombination junctions could be characterized and sequenced directly.

**Figure 2:**
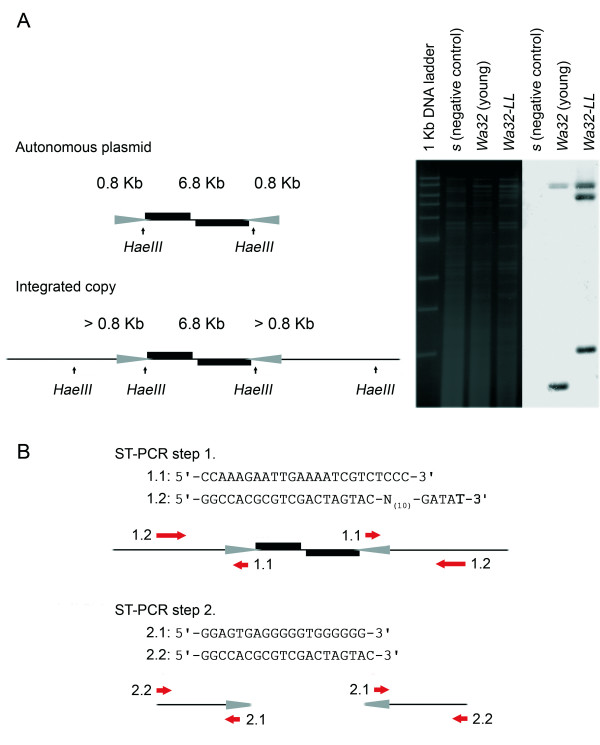
**'Plasmid-tagging' via semi-random two-step PCR (ST-PCR)**. **A**. Detection of integrated pAL2-1 homologues by RFLP analysis. An example is shown of ethidium-bromide stained *HaeIII *digested mtDNA and the corresponding Southern hybridized with the large *XbaI*-3 fragment of pAL2-1. **B**. Amplification of plasmid flanking sequences using semi-random two-step PCR (ST-PCR). In the first round of amplification (using primers 1.1 and 1.2), an 'adaptor' sequence is incorporated. In the second round (using primers 2.1 and 2.2), the flanking sequences are amplified.

Using a modification of a 'plasmid-tagging' method also known as ST-PCR [22] (see Figure 2), we amplified plasmid/mtDNA recombination junctions from senescent cultures. We first tested the validity of the method by employing ST-PCRs on total DNA from young cultures (*i.e*. two or three days old) lacking integrated copies. This served as a negative control. These reactions did not yield any products, even though the cultures carried substantial amounts of the autonomously replicating plasmid (In an approximately 1:1 ratio relative to the mitochondrial genome or superior to that; see Figure 2). We then tested the method by performing an ST-PCR on total DNA from strain *Wa32LL*, which is homoplasmic for an integrated copy upstream of the *nd2 *gene [21]. This served as a positive control. It yielded two products as expected (one for each side of the integrated plasmid copy). Sequencing showed that these products corresponded to the correct 5' and 3' flanking sequences. We concluded that it would be unlikely for junctions thus found to be an artefact of the ST-PCR method.

ST-PCR products obtained from senescing cultures were gel-purified, cloned, sequenced and compared with the mitochondrial genome sequence of *P. anserina *[23, GenBank: X55026]. This yielded twenty plasmid/mtDNA or plasmid/plasmid recombination junctions, among which were 14 unique sites. A summary of these is given in Table 1.

**Table 1:** Plasmid/mtDNA recombination junctions

**#**^**a**^	**Alignment**^**b**^	**Location**^**c**^	**Position**^**d**^
01.		*lrn-i1* (1x)	3,919–21
02.		*orf p'* (1x)	28,808–12
03.		*nd1-i4* (1x)	98,017–25
04.		*nd1-i4* (3x)	98,017–25
05.		between *trna-cys *and *co1* (1x)	38,434–42
06.		*nd3-i1* (1x)	14,362–67
07.		*cob-i1* (1x)	32,164–70
08.		between *orf c *and *atp6* (1x)	22,656–60
09.		between *orf c *and *atp6* (1x)	22,979/80
10.		between *orf c *and *atp6* (2x)	23,009/10
11.		pAL2-1 homologue, *ddrp* (3x)	3,021/22
12.		pAL2-1 homologue, *ddrp* (2x)	2,599/00
13.		*orf p'* (1x)	28,805/06
14.		between *trna-met2 *and *nd2* (1x)	11,337–39
15.		between *trna-met2 *and *nd2* (1x)	11,555–57

**a**. Four of the recombinant sequences (numbers 1, 5, 10 and 14) correspond to 3' plasmid/mtDNA recombination junctions; nine of them (numbers 2–4, 6–9, 13 and 15) to 5' plasmid/mtDNA recombination junctions. Note the MUSE1 undecamer (underlined) found at integration site number 10. Junctions from strain *Wa30 *(numbers 1–12) were found under DR conditions and analyzed by ST-PCR; those from *Wa32 *(numbers 13 and 14) were found under regular conditions; the one from *Wa32-LL *(number 15) had already been sequenced using regular methods [21].

**b**. Alignments of recombinant plasmid/mtDNA and corresponding wild-type sequences, with plasmid-derived parts (corresponding to the 3' terminus of the plasmid) indicated in grey and potential homology in dark grey.

**c**. Locations where the plasmid/mtDNA recombination junctions were found, with the number of times particular junctions were found indicated in brackets.

**d**. Positions correspond to the mitochondrial genome sequence of *P. anserina *laboratory strain *s *[23, GenBank: X55026] or to the sequence of the prototypic pAL2-1 plasmid [8, GenBank X60707].

### The distribution of plasmid integration sites is biased

**Figure 3:**
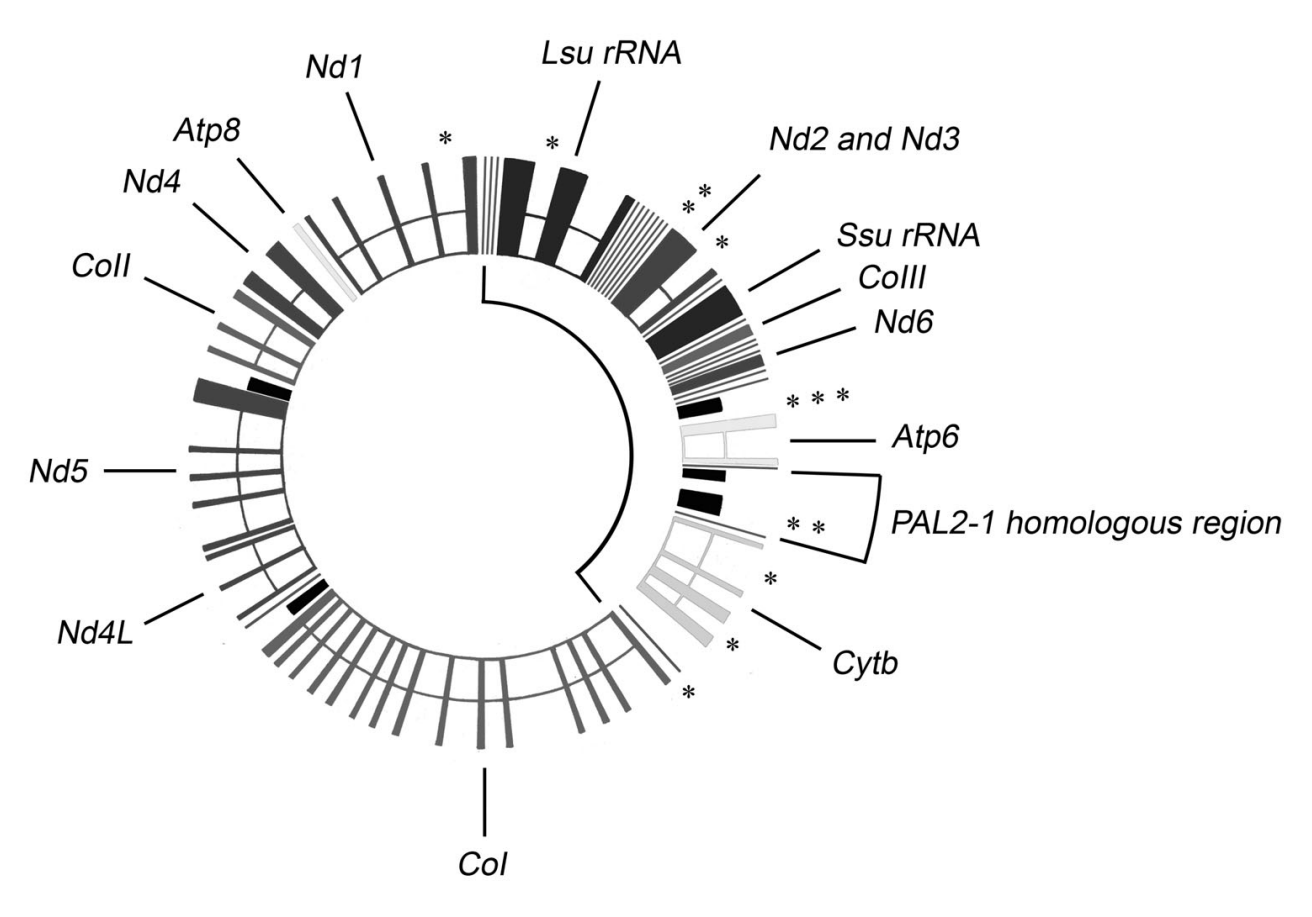
**Integration sites of pAL2-1 homologues in the mitochondrial genome**. Physical map of the ~100 Kb mitochondrial genome of *P. anserina*, indicating the location of plasmid integration sites (asterisks, see Table 1). Integration sites are clustered (Rayleigh's test Z = 6.59, df = 1, P < 0.001; Rao's spacing test U = 188.38, df = 1, P < 0.001). The region affected by integration approximately corresponds to the 37 Kb region that is deleted in 'premature death' strains (indicated in the inner circle). The same region contains a sequence with homology to pAL2-1.

Integrated copies were found in a range of locations. Two of them were found even within a plasmid copy itself. Of those integrated into the mitochondrial genome, all were located within one particular sector (with a mean vector μ of 61.1° ± a circular standard deviation of 44.4°, corresponding to an average position of 17,031 ± 12,361 in the sequence of the mitochondrial genome [23] (see Figure 3). This bias was highly significant (Rayleigh's test; Z = 6.59, df = 1, P < 0.001; Rao's test; U = 188.38, df = 1, P < 0.001). Potential annealing sites of the partially degenerate primer (Figure 2, primer 1.2) were uniformly distributed (Rayleigh's test, Z = 1.283, df = 1, P > 0.05; Rao's test, U = 129.4, df = 1, P > 0.05). We hence concluded that the bias was not an artefact from the PCR-based method, but probably resulted from a true biological phenomenon.

### Plasmid integration is based on a non-homologous mode of recombination

The termini of integrated plasmid copies were often truncated. Also, they often showed short sequence homology (SSH) to the mitochondrial genome (Table 1). This would support the SSH model of integration, were it not that half the integrated plasmid copies were complete and showed no indication of homology with the mtDNA target sequence. DNA sequence homology is therefore not a strict prerequisite for plasmid integration. As discussed further on, junctions with truncated plasmid copies probably resulted from secondary, post-integrative, recombination events. Notably, one of the junctions (Table 1, case 10) coincided exactly with an element known as MUSE1 (for Mitochondrial Ultra-short Sequence Element), as did the secondary recombination junction found in strain *Wa32LL *(see Figure 3). As discussed further on, MUSE1 is frequently observed at recombination junctions in mtDNA from senescent cultures [24,25], and perhaps serves as a recognition sequence for the mitochondrial recombination machinery.

### Integrated plasmid copies are flanked by *de novo *generated mtDNA repeats

**Figure 4:**
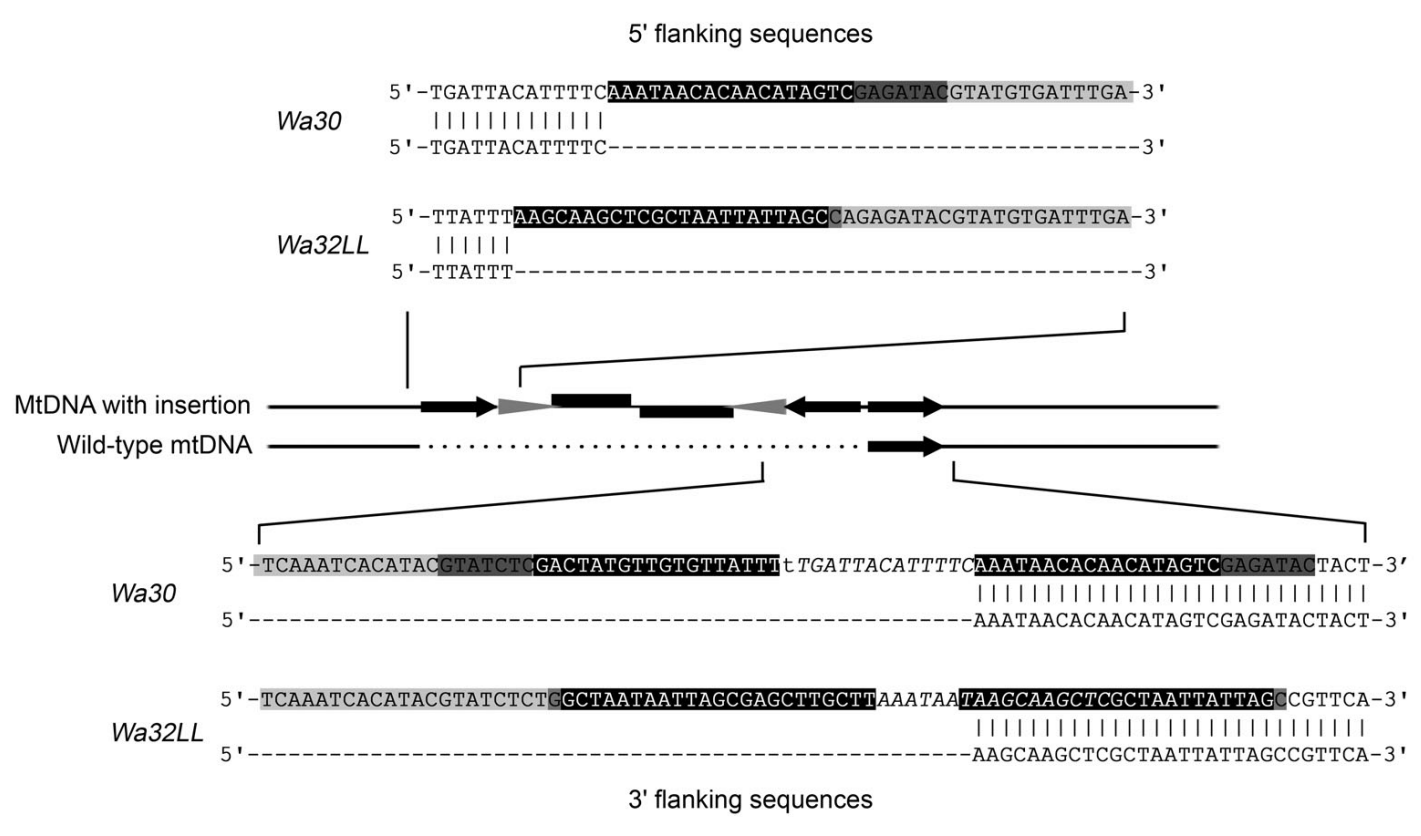
**Plasmid integration results in *de novo *generated mtDNA inversions**. An alignment is given of the 5' and 3' plasmid flanking sequences from the integrations in *Wa30 *and *Wa32-LL *with wild-type mtDNA regions from juvenile cultures of the corresponding strains. As in Table 1, plasmid sequences are indicated in grey, with potential homology to the mitochondrial sequences in dark grey. Duplications are indicated in black. The actual duplications are slightly larger than thus indicated (*i.e*. both in *Wa30 *and in *Wa32-LL *one of the 3' duplications extends further than the other, indicated in italics). Note the linker at the secondary recombination junction of *Wa30 *(small font).

RFLP analyses previously indicated that integrated plasmids of the invertron-type are flanked by long inverted repeats of mtDNA [20,26]. These may result from secondary events involving the 'racquet'- or 'panhandle'-shaped replication intermediates of these plasmids. In our analysis, ST-PCR often (in approximately 90% of the cases) gave only one product whereas at least two were expected. Albeit indirect evidence, this could reflect the presence of inverted repeats. We analyzed the mtDNA sequences on either side of one of the integrated copies and compared these to the corresponding wild-type mtDNA sequence from a young culture. Short direct and inverted duplications were found (see Figure 4). These duplications were not present in young cultures, indicating that they came about *de novo*. We designed specific primers to also amplify the corresponding 5' and 3' flanking mtDNA sequences of other integrated copies described in Table 1. In most cases, the corresponding 5' or 3' recombination junction could not be found. This probably means that the majority of integrated copies is flanked by longer repeats (*i.e*. extending beyond the approximate maximum, several hundreds of base pairs, that can be found using ST-PCR). From RFLP data, previous authors [15] concluded that the inverted repeats may be very large (Their exact endpoints have never been determined), often exceeding at least several Kbs in size, which would be consistent with this observation. The organization of the integration site of the plasmid found in strain *Wa30 *was similar to that found in strain *Wa32-LL*, and it is therefore probably not related to the different phenotypes of these two strains (*i.e*. short- and long-lived, respectively).

## Discussion

PAL2-1 homologous mitochondrial plasmids appear to interfere with DR mediated life span extension [7]. We show that these plasmids act as insertional mutators of the mitochondrial genome, which may directly explain their negative effect on DR mediated life span extension. Using ST-PCR [22] and sequencing, we characterized several different integration sites of the plasmids. We show that the primary integration process is region specific. We also show that it does not necessarily involve short sequence homology, in contrast to the integration process of pKALILO from the related genus of Neurospora. As discussed below, the sequences at and surrounding the recombination junctions probably reflect a mixture of primary and secondary, post-integrative events. We propose a model for the mechanism of integration, and discuss its link with the plasmid's negative effect on DR mediated life span extension.

### The mode and specificity of integration by pAL2-1 homologous plasmids

Previous authors [14] proposed that in Neurospora the integration of pKALILO involves short sequence homology (SSH) with the mtDNA target site. This was based on the observation that sequences of target sites matched those within a 20 bp region of the terminus of the plasmid. Concomitantly, integrated copies of pKALILO were truncated. In contrast however, the integrated copy of the prototypic pAL2-1 plasmid found in *P. anserina *strain *AL2 *[8] did not show sequence homology with the target site. Instead, a short linker sequence was found between the integrated copy of pAL2-1 and the mtDNA target site. The integrated copy was truncated. The question thus remained whether the SSH model of integration could ultimately be maintained, and if so, whether it could be generalized to other invertron-type mitochondrial plasmids. In principle there could be several different modes of integration, *e.g*. depending on the type of invertron or on the host itself. It could also be that secondary recombination events obscure the primary structure of integration sites, leading to false inferences on the mechanism of integration. The latter is not unlikely, as the integrated copies we observe are obviously both the intermediate and final products of mutation plus intracellular selection.

In our analysis, some of the recombination junctions could be explained with the SSH model of integration. There were cases in which the sequence of the target site exactly matched that within the terminal region of the TIR of the plasmid. Integrated plasmid copies were then truncated. This was however not always the case. In half the cases the integrated copies were complete, and in these cases they did not show clear sequence homology to the mtDNA target site. Similar complete integration sites can in fact also be found in *Neurospora intermedia *strains carrying pKALILO (MFPM Maas, unpublished data). This shows that DNA sequence homology is definitely not a strict prerequisite for plasmid integration, neither in Podospora nor in Neurospora.

The junctions likely reflect a mixture of primary and secondary, post-integrative, events. Notably, a MUSE1 undecamer was found at one of the recombination junctions. A MUSE1-like undecamer was also found at the secondary recombination junction in the mtDNA from strain *Wa32LL*. This element has been implied in recombination processes on multiple accounts [24,25], and perhaps serves as a recognition sequence for the mitochondrial recombination machinery of Podospora. It may be the equivalent of elements like the *PstI*-palindromes from Neurospora. Interestingly, the presence of these latter palindromes is inversely correlated with that of plasmid-related sequences in the mitochondrial genome, suggesting that they serve a role in eliminating these and other foreign DNA sequences [27].

These observations first of all show that SSH is not required for plasmid integration. The primary integration process is hence probably based on a mechanism other than homologous recombination, *e.g*. on the protein-mediated juxtaposition of the plasmid's TIR and the mtDNA target sequence. Alternatively, there may be multiple modes of integration that operate simultaneously. In any case, because there is no specific integrase function associated with the plasmids, integration is probably best viewed as an error. This was coined also earlier [28]. If not an active process, integration then perhaps occurs because the termini of the plasmids are erroneously recognized as dsDNA breaks (DSBs). Plasmid integration may be the by-product of erroneous or overly active recombinational repair. A similar process is known from hepaDNA viruses [29]. Integration of hepaDNA viruses depends on the production of linear viral dsDNA and occurs at DSBs, presumably via non homologous end-joining (NHEJ). Notably, in the latter system, short deletions or insertions were observed at the viral/host DNA recombination junctions as was the case at plasmid/mtDNA recombination junctions.

The integration process was neither site-specific nor completely random, but region-specific. To be more precise, it was specific for the region spanning and surrounding the mitochondrial rDNA loci. The integration of pKALILO was originally suggested to have the same specificity [12,13], though from more extensive RFLP data it was later inferred to be random [15]. First of all, the question is to what extent this bias reflects the primary process. It could be that this bias reflects secondary recombination processes. Since we show that secondary recombination processes are probably involved, this is an important question. The bias could for example reflect a progressive deletion of the mitochondrial genome around an origin of replication situated at or near the rDNA loci. This is however probably not the case, as the bias remains when filtering out the integrated copies that may have undergone secondary events (i.e. those that were incomplete). Hence, the observation bias is best explained by a biologically significant bias, *i.e*. one in the primary integration process itself. The region affected could for example be more susceptible to recombination. Perhaps the rDNA region is more heavily transcribed and more accessible to integration. Viral integration is certainly known to be sensitive to the transcriptional state of the targeted locus [30], and this may also be the case for the integration of linear mitochondrial plasmids.

Interestingly, the region affected by plasmid integration corresponds to the same region that is deleted in *AS1-4 *mutant "premature death" strains [31]. The mtDNA deletions in *AS1-4 *are caused by at least two co-existing mechanisms [31]: One that involves the retro-transposition of the group II intron *cox1*-i1 (referred to as a 'class I' mechanism), and one that involves illegitimate recombination (referred to as a 'class II' mechanism). Deletions generated by these two mechanisms occur at a low but constitutive level also in wild-type strains [31], and may correspond to processes involved in mtDNA repair. DSBs can notably be detected *in vivo *in this region [32].

### Integration of pAL2-1 homologues and the life span extending response to DR

PAL2-1 was originally considered a longevity plasmid [19], later a neutral plasmid [6] and more recently a senescence plasmid [7]. This can be explained by the fact that other senescence processes normally overshadow the effects of 'plasmid-based' senescence in Podospora, so that only those cases come to light in which the integration of plasmids is associated with longevity. Under DR conditions however, the pAL2-1 homologues behave like typical senescence plasmids. This is probably best explained directly by their effect on mitochondrial function. Also the integration of senescence plasmids from Neurospora seems to decrease life span rather than increase it. Perhaps the presence of integrated copies thus simply abrogates life span extension via a mechanism that is independent of 'regular' senescence.

It could also be that DR induces a response that makes strains more susceptible to integration events or their effects. As mentioned in the introduction section, DR appears to act by adjusting cellular metabolism to a mode set for survival, enhancing an array of cellular repair systems and general stress responses. First of all, DR may enhance the likelihood of integration events. If integration is indeed a by-product of the erroneous recognition of linear plasmid DNA termini as DSBs, the up-regulation of mitochondrial repair systems would make integration more likely under DR conditions. It must be noted though, that integration also occurs under regular conditions. Secondly, DR may further enhance the effects of integration events. 'Plasmid-based' senescence seems to be a form of induced necrosis [16], a defence response that serves to sequester parts of the host that suffer from the genotoxic effects of, in this case, the integrative plasmids. Enhancement of this general defence response might result in a positive feedback loop, further enhancing the negative effects of the plasmids under DR conditions.

## Conclusion

We have shown that the pAL2-1 homologues act as mitochondrial mutators. They integrate via a non-homologous or mixed mode of recombination and do so in a region-specific manner. Although it is not clear whether their effect on the response to DR is simply due to an abrogation of life span extension via a mechanism that is independent of 'regular' senescence, or via one that is linked to DR-induced host responses, it is clear that 'plasmid-based' senescence in *P. anserina *is similar to that observed in the Neurospora genus. We conclude that plasmid-based senescence in *P. anserina *is probably best viewed as a lethal disease that exerts its effect in the 'shadow' of 'regular' fungal senescence.

## Methods

### Strains and culturing conditions

Isolates *Wa30 *and *Wa32 *are two pAL2-1 homologue carrying *P. anserina *isolates from a collection of Dutch wild types [6,7]. Plasmid-free derivatives of these were obtained by selecting plasmid-free progeny from self-fertilized cultures [7]. *Wa32LL *is a long-lived derivative of *Wa32 *[21].

Cultures were grown at 27°C, at a relative humidity of approximately 70%, on a semi-synthetic (cornmeal) medium with a regular or 100-fold reduced level of glucose [7] as indicated in the text. They were grown either in 50 cm long glass race tubes or on large Petri-dishes of 15 cm in diameter.

### DNA extraction and Southern analysis

Mycelium was harvested from Petri-dish grown mycelia [33], and ground using liquid nitrogen, followed by a standard phenol/chloroform based DNA extraction [34]. Electrophoresis and blotting were done using standard procedures [34]. Blots were probed with a [α-32P] dCTP labelled *XbaI*-3 fragment of pAL2-1 [35].

### ST-PCR based 'plasmid-tagging'

The identification of plasmid integration sites was based on the semi-random two-step PCR (ST-PCR) procedure [22], which is an unbiased way of amplifying unknown flanking sequences. It uses a touchdown PCR to generate fragments with an adaptor sequence, followed by a regular PCR to specifically amplify the fragments containing plasmid/mtDNA recombination junctions. The first step was done using a primer located within the TIR region of the plasmid (primer 1.1: 5'-CCAAAGAATTGAAAATCGTCTCCC-3') in combination with a partially degenerate primer as given in the original protocol (primer 1.2: 5'-GGCCACGCGTCGACTAGTAC-N_(10)_-GATAT-3'). In the first step we used six rounds of touchdown PCR amplification starting with an annealing temperature of 40°C (decreasing by one degree each round), then followed by 24 rounds using an annealing temperature of 60°C. The partially degenerate primer anneals at sites containing the pentanucleotide sequence 5'-ATATC-3', found approximately once every 300 bps throughout the mitochondrial genome. This sequence is distributed uniformly (Rayleigh's test, Z = 1.283, df = 1, P > 0.05; Rao's test, U = 129.4, df = 1, P > 0.05). Hence with regard to the spatial distribution of integration sites found, we inferred that the method of sampling was unbiased. It must be noted though, that the orientation of the pentanucleotide sequence was biased (*i.e*. there were 210 potential forward versus 147 potential reverse priming sites, a significant deviation from the expected 1:1 ratio, likelihood ratio G = 11.18, df = 1, P = 0.001), and the method may have positively biased the amplification of 5' flanking sequences. The second step was done using another primer within the TIR region of the plasmid (primer 2.1: 5'-GGAGTGAGGGGGTGGGGGG-3') and a primer comprising the specific part of the partially degenerate one (primer 2.2: 5'-GGCCACGCGTCGACTAGTAC-3'). In the second step we used 30 rounds of regular PCR amplification and an annealing temperature of 60°C. The principle of 'plasmid-tagging' by ST-PCR is illustrated in Figure 2.

ST-PCR products were gel-purified using a High Pure PCR Product Purification Kit (Roche) and cloned in *E. coli *DH5-α using the pGEM^®^-T Easy Vector system (Promega). Plasmid DNA was recovered using a High Pure Plasmid Isolation Kit (Roche) and sequenced according to the Sanger method, using Big Dye™ terminators (Applied Biosystems). Sequences thus obtained were compared to the mitochondrial genome sequence of *P. anserina *[23], using BLASTn version 2.2.9. [36].

### Statistical analysis

Survival was analyzed using a two-parameter Weibull regression model. Goodness-of-fit was assessed using the Anderson-Darling test.

Distribution data were converted into angular data and analyzed using Rayleigh's uniformity test [37-39] and/or Rao's spacing test [39,40]. This was done using the software Oriana version 2.02 (KCS, Kovach Computing Services). These are single sample tests designed for circular or periodic data, which test the null hypothesis of uniformity against an unspecified alternative. They differ in sensitivity depending on the modality of the underlying distribution: Rao's test may be more sensitive than Rayleigh's one when the distribution is multimodal, because it uses the spacing between successive observations rather than the length of the mean vector.

Frequency data were analyzed using a Likelihood ratio test. This was done using the software SPSS version 11.0 (SPSS).

## Abbreviations

DR: Dietary Restriction, TIR: Terminal Inverted Repeat, SSH: Short Sequence Homology, DSB: Double strands DNA Break

## Authors' contributions

MFPMM participated in the design of the experiments, the data acquisition and statistical analysis, and drafted the manuscript. RFH and AJMD conceived of the study, and AJMD participated in its design and coordination and helped to draft the manuscript. All authors read and approved the final manuscript.
